# 244-MPT overcomes gefitinib resistance in non-small cell lung cancer cells

**DOI:** 10.18632/oncotarget.6236

**Published:** 2015-10-26

**Authors:** Yi Zhang, Ke Yao, Chengcheng Shi, Yanan Jiang, Kangdong Liu, Song Zhao, Hanyong Chen, Kanamata Reddy, Chengjuan Zhang, Xiaoyu Chang, Joohyun Ryu, Ann M. Bode, Ziming Dong, Zigang Dong

**Affiliations:** ^1^ The Hormel Institute, University of Minnesota, Austin, MN, USA; ^2^ Pathophysiology Department, Basic Medical College, Zhengzhou University, Henan, China; ^3^ The First Affiliated Hospital of Zhengzhou University, Henan, China; ^4^ The China-US (Henan) Hormel Cancer Institute, Henan, China; ^5^ The Affiliated Cancer Hospital of Zhengzhou University, Henan, China

**Keywords:** gefitinib resistance, non-small cell lung cancer (NSCLC), epidermal growth factor receptor (EGFR)

## Abstract

The epidermal growth factor receptor (EGFR) is known to play a critical role in non-small cell lung cancer(NSCLC). Several EGFR tyrosine kinase inhibitors(TKIs), such as gefitinib, have been used as effective clinical therapies for patients with NSCLC. Unfortunately, acquired resistance to gefitinib commonly occurs after 6–12 months of treatment. The resistance is associated with the appearance of the L858R/T790M double mutation of the EGFR. In our present study, we discovered a compound,referred to as 244-MPT, which could suppress either gefitinib-sensitive or -resistant lung cancer cell growth and colony formation, and also suppressed the kinase activity of both wildtype and double mutant (L858R/T790M) EGFR. The underlying mechanism reveals that 244-MPT could interact with either the wildtype or double-mutant EGFR in an ATP-competitive manner and inhibit activity. Treatment with 244-MPT could substantially reduce the phosphorylation of EGFR and its downstream signaling pathways, including Akt and ERK1/2 in gefitinib-sensitive and -resistant cell lines. It was equally effective in suppressing EGFR phosphorylation and downstream signaling in NL20 cells transfected with wildtype, single-mutant (L858R) or mutant (L858R/T790M) EGFR. 244-MPT could also induce apoptosis in a gefitinib-resistant cell line and strongly suppress gefitinib-resistant NSCLC tumor growth in a xenograft mouse model. In addition, 244-MPT could effectively reduce the size of tumors in a gefitinib-resistant NSCLC patient-derived xenograft (PDX) SCID mouse model. Overall, 244-MPT could overcome gefitinib-resistance by directly targeting the EGFR.

## INTRODUCTION

Lung cancer is one of the most common cancers in the United States. In 2015, approximately 240,000 cases will be diagnosed [1]. Non-small cell lung cancer (NSCLC) is the major type of lung cancer (87%) and the overall 5-year survival rate for NSCLC is only 18.2% [2]. The epidermal growth factor receptor (EGFR) reportedly plays a critical role in NSCLC [3], with about 40–80% of NSCLC patients exhibiting elevated EGFR expression [4]. The RAS/RAF/MEK/ERKs and the PI3-K/Akt pathways are two primary signaling networks activated by EGFR [5, 6]. These pathways drive tumor cell proliferation, survival, invasion and angiogenesis [7]. In NSCLC, mutations are frequently observed in the EGFR [8]. Classical-activating mutations include deletions in exon 19 and a point mutation of L858R, which constitute about 90% of all EGFR-activating mutations [9].

Because of the crucial role of EGFR in tumorigenesis, several EGFR tyrosine kinase inhibitors (TKIs), including gefitinib and erlotinib, have been developed as effective clinical therapies for patients with activating mutations. The magnesium-ATP-binding pocket of the intracellular tyrosine kinase domain can be blocked by TKIs, which means that the ligand-induced receptor auto-phosphorylation function is obstructed by the binding of a TKI to the tyrosine kinase domain. This binding disrupts tyrosine-kinase activity, thereby inhibiting intracellular downstream signaling [10]. Unfortunately, acquired resistance to the TKI often manifests within 6 to 12 months of treatment [11]. Patients with acquired resistance to gefitinib or erlotinib exhibit a secondary mutation of EGFR in exon 20, which leads to a substitution of methionine for threonine at position 790 (T790M) in the kinase domain [12]. Thr790 of the EGFR is considered to be a ‘gatekeeper’ residue, which is an important determinant of inhibitor specificity in the ATP-binding pocket of the EGFR [10]. Research results have shown that the T790M mutation causes resistance to TKIs by increasing the affinity of EGFR for ATP [7, 10]. Therefore, finding a drug that can inhibit the double mutant EGFR (L858R/T790M) to overcome gefitinib resistance in NSCLC treatment is greatly needed. By using computational methods to screen for small molecule inhibitors in the ZINC natural compound database, we identified 244-MPT (2-[4-(4-methoxyphenoxy)-1H-pyrazol-3-yl]-5-(*p*-tolylmethoxy)phenol), as a potential EGFR inhibitor that could be used in NSCLC therapy. We found that this compound could suppress gefitinib-sensitive and -resistant lung cancer cell growth by inhibiting EGFR activity and its downstream signaling pathways *in vitro* and *ex vivo*. Moreover, we observed that 244-MPT could strongly suppress gefitinib-resistant lung cancer tumor growth in both a mouse xenograft model and a PDX model.

## RESULTS

### 244-MPT, inhibits anchorage-independent growth and proliferation of both gefitinib-sensitive and -resistant lung cancer cells

**Figure 1:**
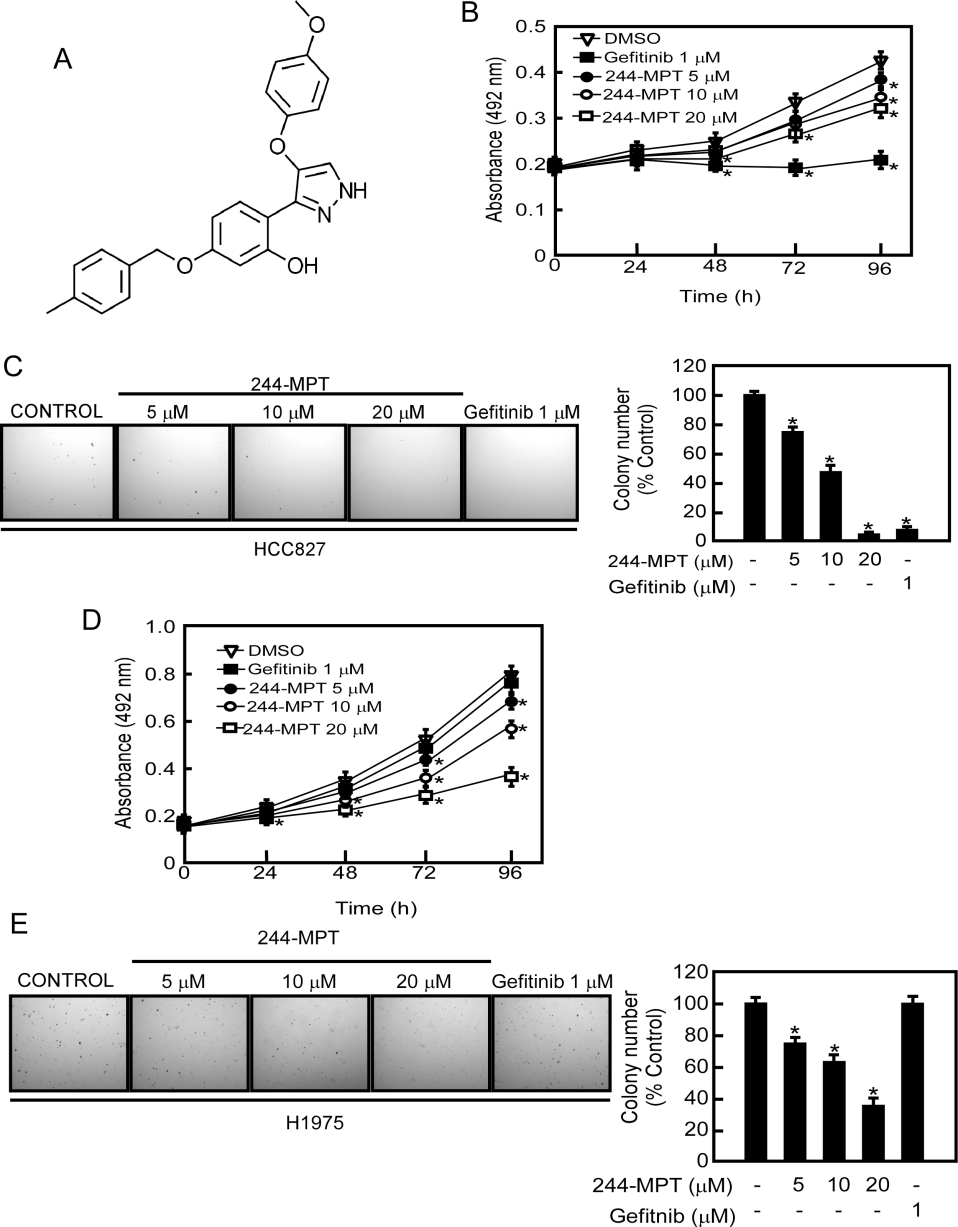
244-MPT suppresses anchorage-independent soft agar growth and decreases viability of either gefitinib-sensitive or -resistant NSCLC cell lines (**A**) Chemical structure of 244-MPT. (**B**) HCC827 or (**C**) H1975 lung cancer cells were treated or not treated with different concentrations of 244-MPT or gefitinib for 24, 48, 72 or 96 h and proliferation was estimated by MTS assay. (**D**) HCC827 or (**E**) H1975 lung cancer cells were treated with increasing doses of 244-MPT or with gefitinib (1 μM). Representative photographs are shown and data are presented as mean values ± S.D. from triplicate experiments. The asterisk (*) indicates a significant (*p* < 0.05) decrease in colony formation compared to the control group.

Over 50% of non-small cell lung cancer (NSCLC) patients exhibit elevated EGFR expression. Notably, most lung cancer patients expressing the T790M EGFR mutation respond to initial gefitinib treatment but eventually acquire resistance [13]. Thus, identifying a drug that can overcome gefitinib resistance in NSCLC is crucial. 244-MPT (Figure 1A) was identified through our computational screening as a novel compound that might target both wildtype and mutant EGFR (L858R/T790M). To determine whether 244-MPT exerted any cytotoxic effects against normal lung cells, MRC-5 cells, a human normal lung fibroblast cell line, were treated with different concentrations of 244-MPT for 24 or 48 h. The results indicated that 244-MPT had no cytotoxicity at concentrations less than 40 μM (Supplementary Figure 1). Next, human H1975 lung cancer cells, which are gefitinib-resistant, and HCC827 gefitinib-sensitive lung cancer cell lines, were treated with different doses of 244-MPT. Our results revealed that gefitinib [14] and 244-MPT strongly inhibited gefitinib-sensitive HCC827 cell proliferation and colony formation (Figure 1B, 1C). Notably, 244-MPT also markedly suppressed H1975 gefitinib-resistant cell proliferation and colony formation dose-dependently, whereas gefitinib was ineffective (Figure 1D, 1E). These data reveal that 244-MPT might be a potential molecule to overcome gefitinib resistance in NSCLC.

### 244-MPT binds and inhibits both wildtype and mutant EGFR activities *in vitro* and *ex vivo*

**Figure 2:**
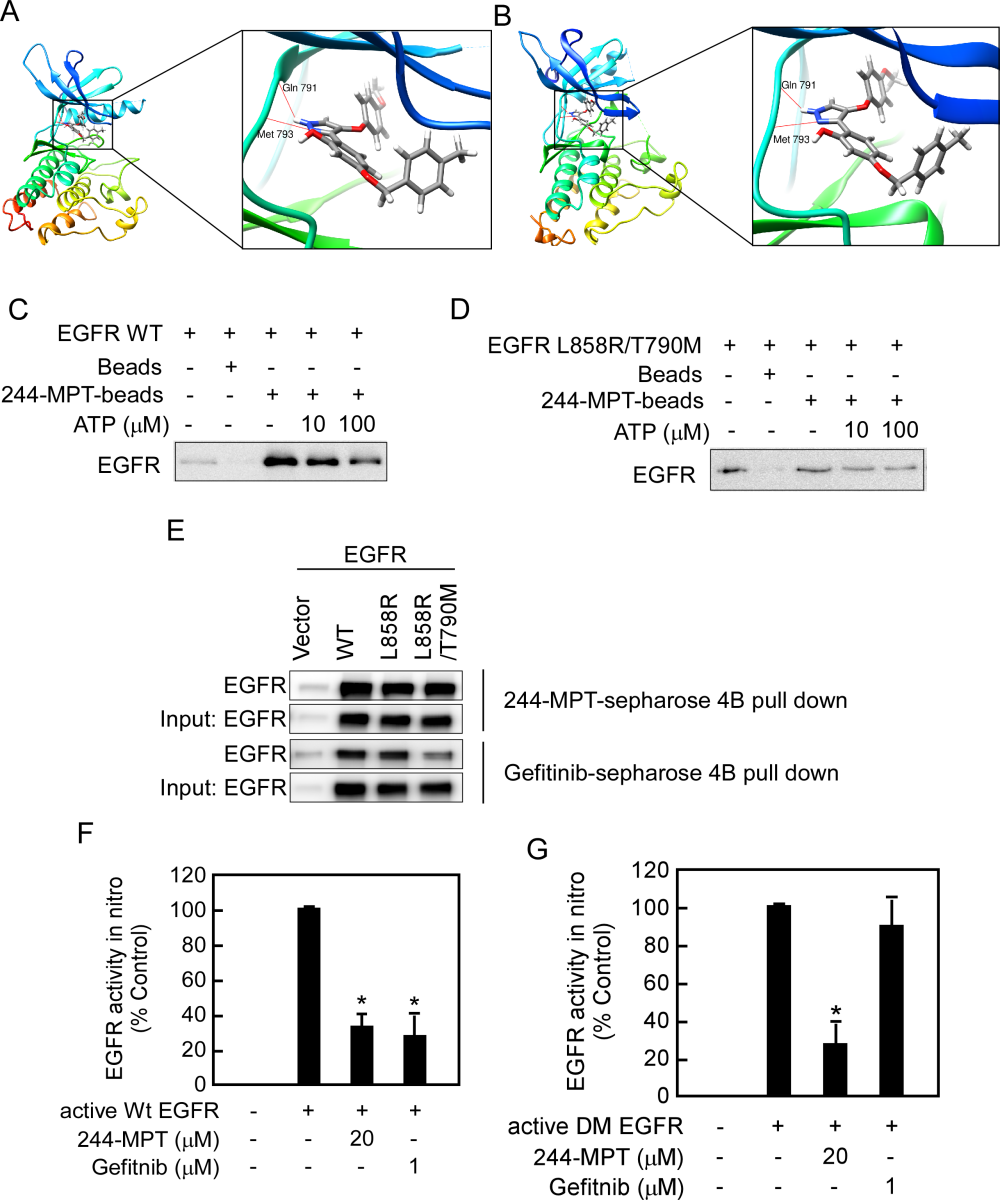
244-MPT binds and inhibits both wildtype and mutant EGFR activities *in vitro* and *ex vivo* Computational docking model of 244-MPT with (**A**) wildtype EGFR or (**B**) double mutant (L858R/T790M) EGFR. Pull-down assays were used to confirm (**C, D**) *in vitro* ATP competitive binding or *ex vivo* (**E**) binding of 244-MPT with wildtype or mutant EGFR. A recombinant human EGFR protein or 293T cell lysates and different concentrations of ATP were incubated with 244-MPT-Sepharose 4B, gefitinib-Sepharose 4B, or only Sepharose 4B beads. Proteins were immunoprecipitated with an EGFR antibody and then subjected to Western blotting to examine the interaction between 244-MPT and EGFR. Data are representative of 3 separate experiments eliciting similar results. (**F**) Active wildtype EGFR or (**G**) active double mutant (L858R/T790M) EGFR was incubated with 244-MPT (20 μM) or gefitinib (1 μM) with angiotensin II as substrate and a [γ-^32^P] ATP mixture. The incorporated radioactivity was determined using a scintillation counter. Data are represented as mean values ± S.D. from 3 independent experiments. The asterisk (*) indicates a significant (*p* < 0.05) decrease in kinase activity compared to untreated control.

To better understand the mechanism of the interaction of 244-MPT with wildtype and mutant EGFR, we conducted an *in silico* docking assay. The computational binding models showed that several hydrogen bonds were formed between 244-MPT and the EGFR ATP pocket in either the wildtype or mutant protein (Figure 2A, 2B). To further confirm our result, we conducted an energy minimization and molecular dynamics (MD) simulation (Supplementary Videos S1 and S2). We compared the binding mode changes after 5 ns MD of 244-MPT and gefitinib. Results showed that after 5 ns MD, 244-MPT still formed some hydrogen bonding, hydrophobic and other interactions with double mutant EGFR (L858R/T790M), whereas gefitinib did not form hydrogen bonds, but only the hydrophobic and other interactions with double mutant (L858R/T790M) EGFR (Supplementary Figure 2). This result indicated that 244-MPT can bind with double mutant (L858R/T790M) EGFR tightly. An ATP competition assay further showed that either wildtype (Figure 2C) or mutant EGFR (Figure 2D) was pulled down by 244-MPT-conjugated Sepharose 4B beads but not Sepharose 4B beads alone. Moreover, the binding ability of 244-MPT with either EGFR wildtype or mutant was reduced in the presence of ATP (Figure 2C, 2D). Furthermore, we observed *ex vivo* binding between 244-MPT and EGFR in 293T cells overexpressing exogenous wildtype or mutant EGFR (Figure 2E). 244-MPT could strongly bind with wildtype, the L858R single mutant EGFR or the L858R/T790M double mutant EGFR, whereas the binding affinity between gefitinib and the double mutant EGFR was much weaker (Figure 2E). To elucidate the effect of 244-MPT against EGFR wildtype and mutant activity, we conducted *in vitro* kinase assays. As predicted, either 244-MPT or gefitinib strongly inhibited wildtype EGFR kinase activity (Figure 2F), whereas 244-MPT also substantially inhibited the kinase activity of the EGFR L858R/T790M double mutant while gefitinib was ineffective (Figure 2G). All these results indicate that 244-MPT interacts with wildtype or mutant EGFR at the ATP-binding pocket and inhibits their respective kinase activity.

### 244-MPT attenuates EGFR signaling in both gefitinib-sensitive and -resistance NSCLC cells

**Figure 3:**
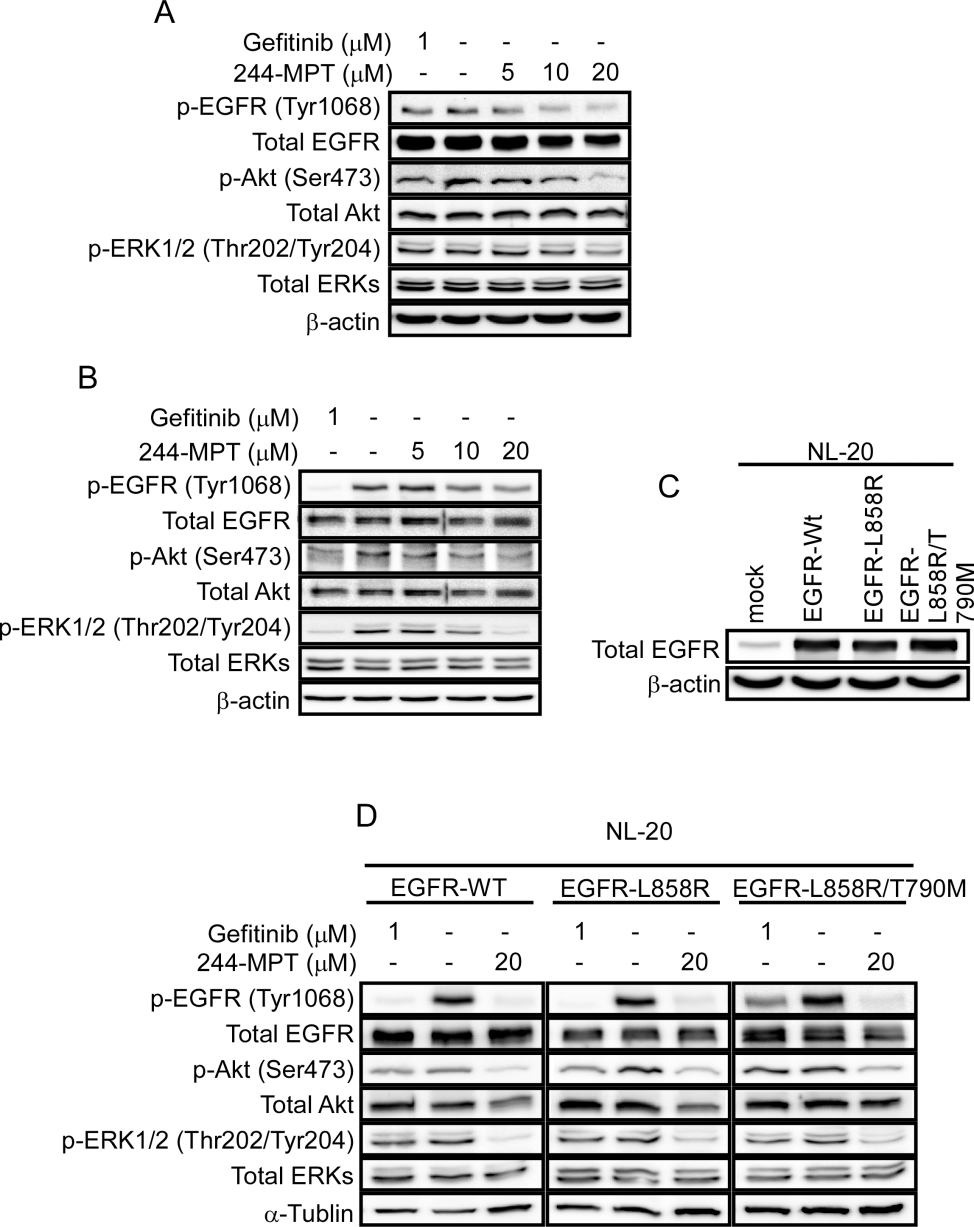
244-MPT inhibits EGFR signaling (**A**) HCC827 or (**B**) H1975 cells were treated with different concentrations of 244-MPT or gefitinib for 24 h. Cells were harvested and total EGFR, Akt, ERK1/2 and phosphorylated EGFR, Akt, ERK1/2 and β-actin were detected by Western blotting. (**C**) Expression level of EGFR in EGFR-transfected NL20 stable cell lines. (**D**) NL20 cells stably transfected with wildtype EGFR, L858R single mutant EGFR or L858R/T790M double mutant EGFR were treated or not treated with the indicated concentration of 244-MPT or gefitinib. Protein expression was detected by Western blot. Representative blots from 3 independent experiments are shown.

Both the Akt/mTOR and the MAPK cascades are primary downstream pathways of EGFR signaling [15]. We observed persistent phosphorylation of Akt and ERK1/2 despite gefitinib treatment in H1975 cells (Figure 3A 1st lane); whereas gefitinib abolished phosphorylation of Akt and ERK1/2 in the sensitive HCC827 cells (Figure 3B 1st lane). These data suggest the presence of persistently activated EGFR (L858R/T790M)/Akt and EGFR (L858R/T790M)/ERKs cascades in gefitinib-resistant cells. A previous report [10] and our data showed that the T790M mutation reduced the effectiveness of gefitinib to compete for binding at the ATP pocket, but 244-MPT showed much stronger binding affinity compared with gefitinib in either EGFR wildtype or mutant cells (Figure 2E). Thus to further study the effect of 244-MPT, we examined the effect of different doses of 244-MPT on phosphorylation of EGFR, Akt and ERK1/2 in HCC827 and H1975 cells. Western blot results showed that 244-MPT strongly suppressed phosphorylation of EGFR, Akt and ERK1/2 in both gefitinib-sensitive and -resistant NSCLC cells (Figure 3A, 3B). We then established NL-20 cells stably overexpressing wildtype or mutant EGFR (Figure 3C) and examined the effect of 244-MPT. Similar effects of 244-MPT and gefitinib were observed in the NL-20 cells overexpressing wildtype, the L858R single mutant EGFR or the L858R/T790M double mutant EGFR (Figure 3D).

### 244-MPT induces apoptosis in gefitinib-resistant cells

**Figure 4:**
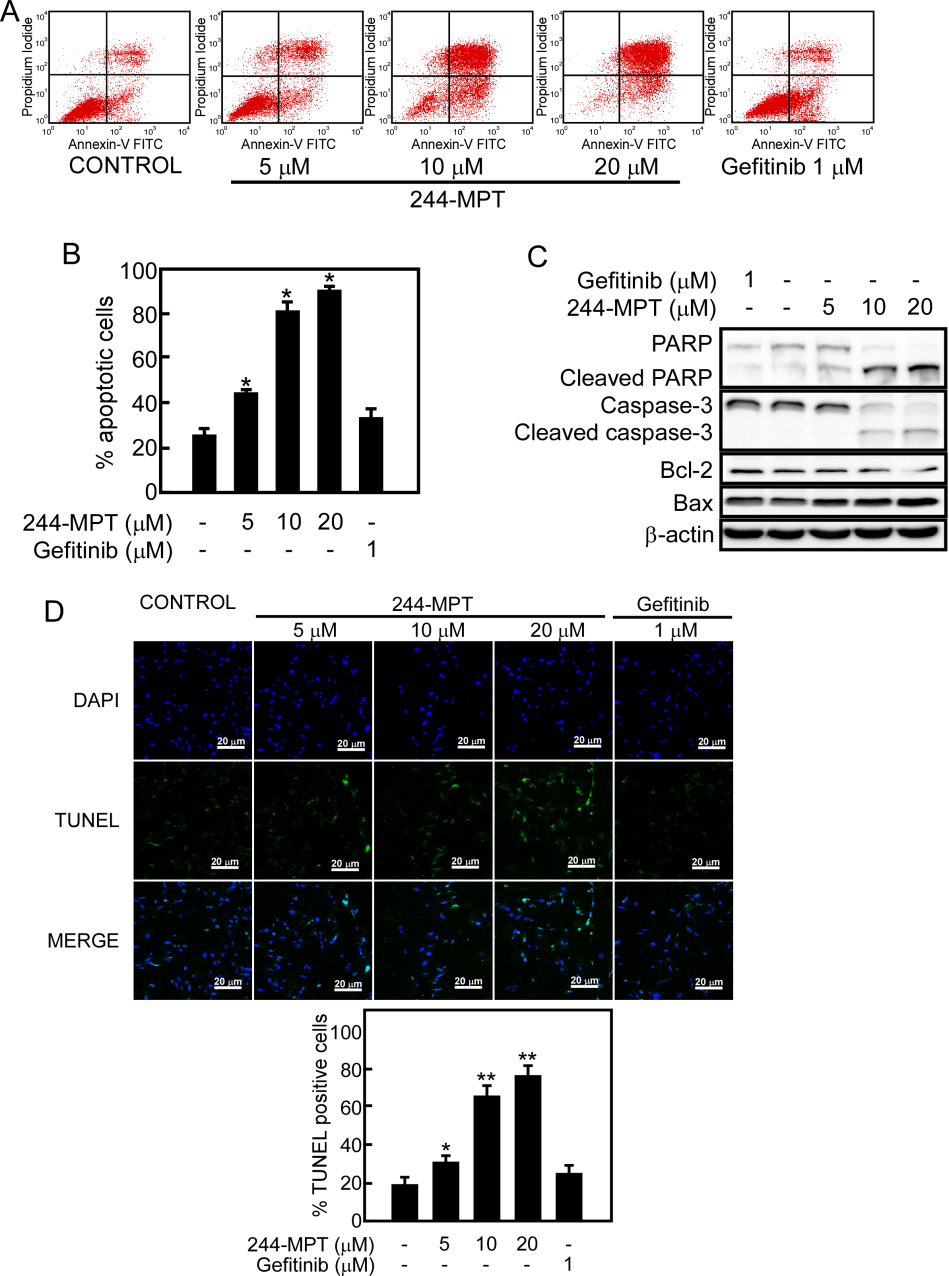
244-MPT induces apoptosis in H1975 cells (**A, B**) H1975 lung cancer cells were treated with different concentrations of 244-MPT or gefitinib for 24 h and then stained with annexin V. Apoptosis was determined by flow cytometry. (**C**) Cells were treated with vehicle, or 5, 10, or 20 μM 244-MPT or 1 μM gefitinib for 24 h. 244-MPT increases pro-apoptotic protein expression and suppresses anti-apoptotic protein expression. Data are representative of 3 independent experiments that gave similar results. (**D**) Visualization of apoptotic cells using the TUNEL assay. H1975 cells were stained with DAB or DAPI after treatment with different concentrations of 244-MPT or gefitinib. Cells not undergoing apoptosis are blue and cells undergoing apoptosis exhibit green nuclear fragments. Data are quantified in lower panel and the asterisks (*, **) indicate a significant increase in TUNEL positive cells (*p* < 0.05, *p* < 0.01).

Induction of apoptosis has been a key strategy for effective cancer therapy. We used Annexin V staining and flow cytometry to further examine whether 244-MPT could induce apoptosis in H1975 gefitinib-resistant cells. Data showed that treatment with 244-MPT caused significant apoptosis in this cell line, whereas gefitinib was less effective (Figure 4A, 4B). In addition, treatment of H1975 cells with 244-MPT induced the cleavage of caspase-3 and PARP, which are markers of apoptosis (Figure 4C). Treatment with 244-MPT increased the expression of the pro-apoptotic protein, Bax, and reduced the expression of the anti-apoptotic protein, Bcl-2 (Figure 4C). The TUNEL assay provided similar results showing that treatment with 244-MPT caused substantially more apoptosis compared to the untreated control or gefitinib treatment (Figure 4D).

### 244-MPT suppresses tumor growth of gefitinib-resistant cells in a xenograft mouse model

**Figure 5:**
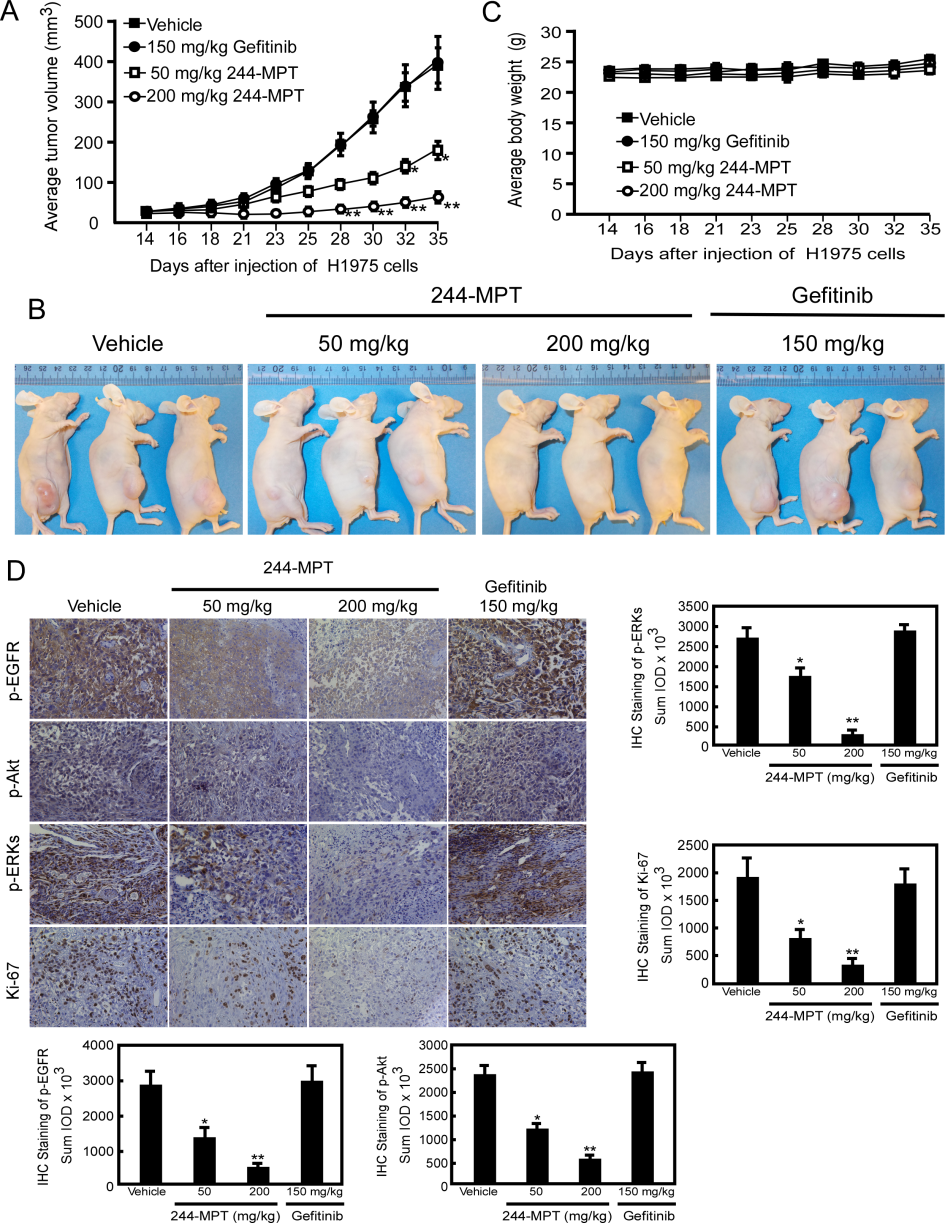
244-MPT inhibits tumor growth in a gefitinib-resistant NSCLC xenograft mouse model (**A**) Treatment with 244-MPT significantly suppresses NSCLC xenograft tumor volume compared with the vehicle-treated group. The asterisks (*, **) indicate a significant (*p* < 0.05, *p* < 0.01, respectively) decrease in tumor volume compared to the vehicle-treated control. (**B**) Representative photographs show external appearance of tumors. (**C**) 244-MPT has no effect on the body weight of mice. (**D**) Immunohistochemistry analysis was used to determine the level of Ki-67, and phosphorylation of EGFR, Akt and ERK1/2 in tumors treated with 244-MPT compared tumors treated with vehicle only. Representative photographs for each antibody and each group are shown. The integrated optical density (IOD) was evaluated using the Image-Pro Premier software offline (v.9.0) program. The asterisks (*, **) indicate a significant (*p* < 0.05, *p* < 0.01, respectively) decrease in compound-treated samples compared to vehicle-treated samples.

For many years, mouse xenograft models implanted with human cancer cells lines have been used extensively for predicting responsiveness to anticancer target agents. Based on our findings thus far *in vitro* and *ex vivo*, we next determined the chemotherapeutic effect of 244-MPT *in vivo.* Using an athymic nude xenograft mouse model, results showed that 244-MPT suppressed H1975-xenograft tumor growth, whereas gefitinib was ineffective in reducing tumor size (Figure 5A, 5B). The highest dose of 244-MPT (200 mg/kg) treatment showed significantly more inhibition compared to the lowest dose (50 mg/kg). After mice were sacrificed, tumors were weighed and these results were confirmed (Supplementary Figure 3A, 3B). The body weights of all animals remained stable after daily treatment by oral gavage with 244-MPT, gefitinib, or vehicle control (Figure 5C), which suggested that the dose of 244-MPT used for the experiment had no toxicity to the mice. To further confirm that the antitumor effect of 244-MPT was associated with its inhibition of EGFR, an immunohistochemical analysis was performed. These results showed that Ki-67 expression and phosphorylation of EGFR, Akt and ERK1/2 were each significantly suppressed in the 244-MPT treated-groups compared with the vehicle- or gefitinib-treated group (Figure 5D). These results clearly indicated that 244-MPT exerts a substantial chemotherapeutic effect to overcome gefitinib-resistant xenograft growth in mice acting mainly through the suppression of EGFR activation.

### 244-MPT suppresses tumor growth in a gefitinib-resistant NSCLC PDX model

**Figure 6:**
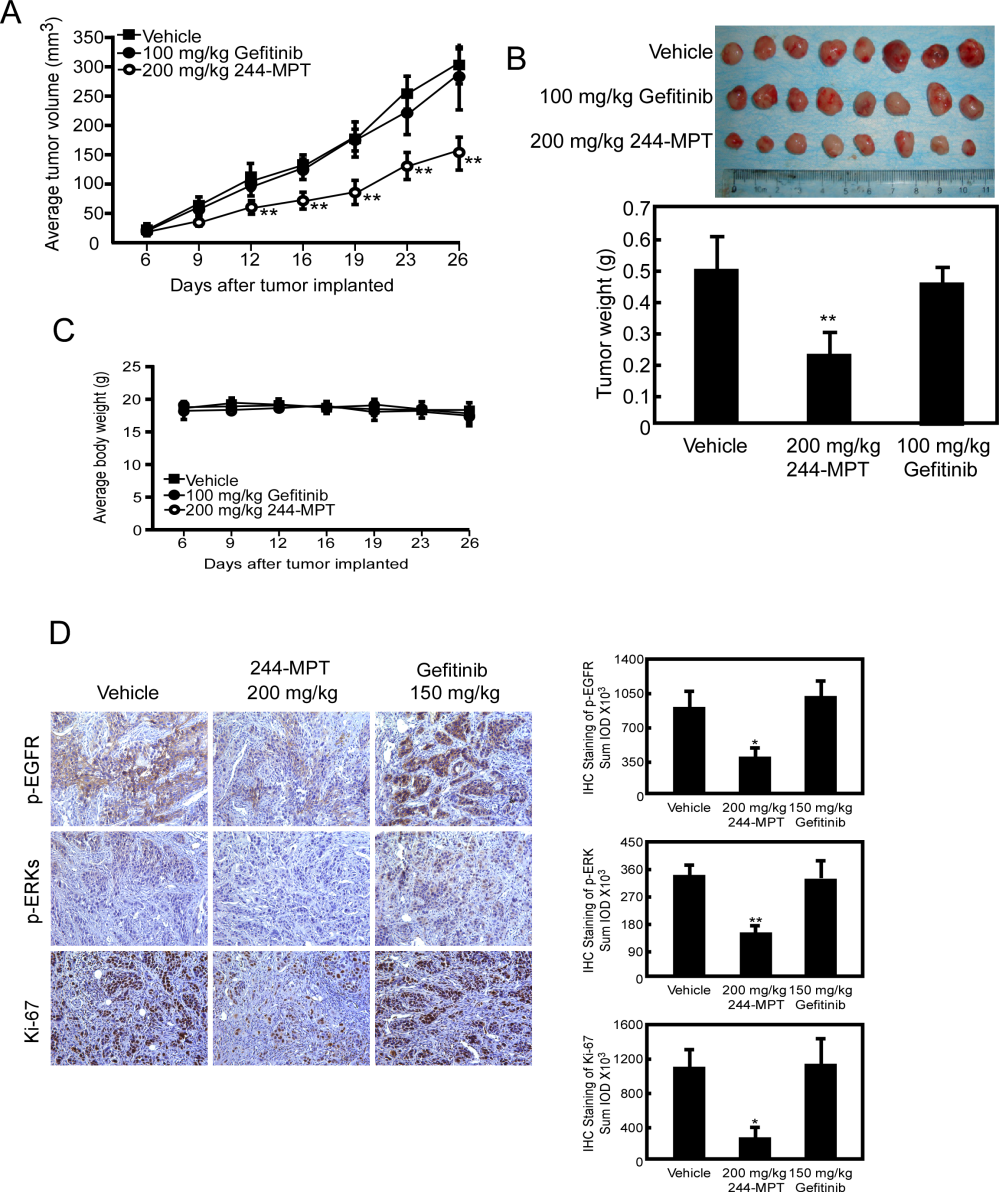
244-MPT inhibits tumor growth in a gefitinib-resistant NSCLC PDX mouse model (**A**) Treatment with 244-MPT significantly suppresses NSCLC PDX tumor volume compared with the vehicle-treated group. The asterisks (**) indicate a significant (*p* < 0.01) decrease in tumor volume compared to untreated control. (**B**) Tumor volume and tumor weight were measured. The asterisk (**) indicates a significant (*p* < 0.01) decrease in 244-MPT-treated mice compared to vehicle-treated mice. (**C**) 244-MPT has no effect on the body weight of mice. (**D**) Immunohistochemistry analysis was used to determine the levels of Ki-67 and phosphorylation of EGFR and ERK1/2 in tumors treated with 244-MPT compared with vehicle-treated. Representative photographs for each antibody and each group are shown. The integrated optical density (IOD) was evaluated using the Image-Pro Premier software offline (v.9.0) program. The asterisks (*, **) indicate a significant (*p* < 0.05, *p* < 0.01, respectively) decrease in compound-treated compared to vehicle-treated samples.

The PDX model is based on the transfer of primary tumors directly from the human patient into an immune deficient mouse. To further investigate the chemotherapeutic effect of 244-MPT and its clinical relevance, we created a model that mimicked a patient who was sensitive to gefitinib, treated with gefitinib for a certain period, and finally followed by the emergence of gefitinib-resistance. In the gefitinib-resistant PDX model, results showed that 244-MPT suppressed gefitinib-resistant NSCLC PDX tumor growth with no toxicity, whereas gefitinib had no effect (Figure 6A–6C). Additionally, an immunohistochemical analysis showed that Ki-67 and phosphorylation of EGFR and ERK1/2 were each significantly suppressed in the 244-MPT treated-groups compared with the vehicle- or gefitinib-treated group (Figure 6D). All these data indicated that 244-MPT might be a very promising novel drug candidate to be evaluated in clinical trials.

## DISCUSSION

Lung cancer is the leading cause of cancer death among males in both highly developed and less developed countries, and has surpassed breast cancer as the leading cause of cancer death among females in more developed countries [16]. An estimated 1.8 million new lung cancer cases occurred in 2012 worldwide, accounting for about 13% of total cancer diagnoses [16]. Non-small-cell lung cancer (NSCLC) is the most common type of lung cancer, accounting for more than 80% of all cases [17]. Approximately 70–75% of patients with NSCLC have advanced stage disease when first diagnosed [18]. Platinum-based chemotherapy is the standard care for patients with advanced NSCLC. Historically, all patients were treated with the same systemic regimens regardless of histological subtype or molecular features [19]. However, chemotherapy is fraught with harmful side effects such as fatigue, myelosuppression, neutropenia, diarrhea and nausea [20]. Moreover, data indicate that the response rate of advanced stage NSCLC patients to current standard combination chemotherapy is only 20–25% and the estimated 2- or 5-year survival rates are only 15- or 2%, respectively [17, 21, 22]. Novel therapies include the targeting of biomarkers, such as EGFR, that are commonly overexpressed in lung cancers. EGFR mutation is identified as a cause of NSCLC and its activation corresponds to poor prognosis [23]. The in-frame deletions in exon 19 and a point mutation that substitutes Leu858 with arginine account for almost of all the activating mutations [24]. These mutations cause constitutive activation of kinase activity by destabilizing the auto-inhibited conformation of the kinase, which initially confers sensitivity to the TKIs. TKIs, like gefitinib and erlotinib, were found to be most effective in patients who harbor somatic activating mutations [25]. TKIs are generally less toxic and more efficacious than standard chemotherapy [26].

Unfortunately, the efficacy of TKI treatment often has limited duration because of the emergence of drug resistance conferred by a second mutation, the substitution of threonine 790 with methionine (T790M), which accounts for about 50% of all resistance to gefitinib and erlotinib [27]. In randomized studies, the median overall survival of patients with EGFR mutant lung cancer receiving first-line EGFR TKIs is 19 to 36 months, while median progression-free survival is about a year. In more than half of patients, tumor cells at the time of progression harbor a second-site T790M mutation, which confers resistance to EGFR TKIs [28]. No specific treatments for patients with acquired resistance to current EGFR TKIs have yet been approved.

We performed computer screening, which designed to specifically target wildtype and L858R/T790M mutant EGFR, using the ZINC natural database and 244-MPT was listed at the top of the screening results. We show that 244-MPT is a novel EGFR inhibitor with a distinct chemical structure from other TKIs (Figure 1A), and it markedly inhibited anchorage-independent growth of both gefitinib-sensitive and –resistant NSCLC cell lines (Figure 1C, 1E). *In silico* docking and *in vitro* kinase assay results (Figure 2) together with *ex vivo* cellular phosphorylation data (Figure 3) collectively showed that 244-MPT is highly potent against both EGFR wildtype and T790M mutant expressing cells. Moreover, the profound anti-tumor activity of 244-MPT across xenografts expressing T790M EGFR *in vivo* suggests a great potential to target T790M-expressing tumors (Figure 5). We also imitated in mice the clinical process of acquiring gefitinib-resistance. Continuous treatment of SCID mice implanted with human gefitinib-sensitive tumors established a gefitinib-resistant NSCLC PDX mouse model. When these mice were treated with 244-MPT, similar results were obtained as for the T790M xenograft mouse model (Figure 6). This result clearly suggests that 244-MPT could effectively reduce gefitinib-resistant patient-derived tumor size. In the PDX model, solid tumor fragments are implanted into immunodeficient mice. This model is considered to be highly relevant to real human tumor growth because it develops a functional stroma and vasculature, microenvironment and displays central necrosis and reflects tumor differentiation. These tumors often maintain the original molecular characteristics and heterogeneity of the original patient tumor better than tumors generated from transplanted cell lines. Many factors, such as angiogenesis, growth factors, stroma and microenvironment can affect tumor growth and the effect of a drug. This explains why PDX data are more convincing than single cell line xenografts. Thus, the effect of 244-MPT on tumor growth might be affected by these factors but its effect on its targeted markers could very likely be similar in cells and whole tumors. The PDX model has strong predictive power for translating preclinical efficacy into clinical outcome [29]. Although 244-MPT exhibited a little less inhibitory effect in the PDX model compared to the gefitinib-resistant H1975 cell line xenograft model, but still strongly inhibited gefitinib-resistant patient-derived tumor growth, this result supported 244-MPT as a novel and effective drug, providing a good foundation for further study.

The T790M mutation confers drug resistance by increasing the ATP affinity of the oncogenic L858R mutant EGFR by more than an order of magnitude, thereby reducing the effectiveness of TKIs to compete for binding at the ATP pocket [10, 28]. Our computational prediction and pull-down assay results clearly confirmed that 244-MPT could bind to either the wildtype or T790M EGFR (Figure 2). To support our hypothesis, computational simulation results showed that after performing a 5 ns MD simulation, 244-MPT, but not gefitinib, still binds tightly with the double mutant EGFR, 4 (Supplementary Figure 2). With the aid of supercomputers, the MD simulation could be produced easily and provided more evidence explaining our results. The EGFR transmits activating signals to various downstream signaling molecules and the two main pathways include the PI3-K/Akt and MAPK pathways. Previous reports show that the T790M mutation activates the kinase activity of EGFR at least 5-fold compared with the wildtype enzyme [10]. Our results indicated that 244-MPT effectively attenuated the phosphorylation of Akt and ERK1/2 in both wildtype and T790M mutant lung cancer cells and also reduced their viability and increased apoptosis compared with the traditional TKI, gefitinib (Figures 3, 4). Up to 20% of EGFR-mutated lung cancers that have developed acquired resistance to TKIs also exhibit MET amplification in this setting [30, 31]. When patients acquire resistance and harbor both T790M mutation and MET amplification, a c-MET inhibitor alone is not effective [32]. MET amplification can lead to persistent activation of the PI3-K/Akt signaling pathway, contributing to tumorigenesis [27]. The inhibition of EGFR wildtype and T790M mutant by 244-MPT strongly suppresses Akt activation, suggesting that 244-MPT might also affect MET [30, 31]. We will study this in more detail in the future.

Clearly, finding an agent that can more effectively target the T790M EGFR mutant for advanced or recurrent NSCLC is critical. Our results strongly support the idea that 244-MPT could act as an effective EGFR wildtype and T790M inhibitor that can overcome TKI resistance in NSCLC treatment, which could then lead to delayed disease progression and ultimately increased survival benefit.

## MATERIALS AND METHODS

### Chemicals and reagents

244-MPT was synthesized in-house following a protocol reported for similar compounds [33]. Gefitinib was from AstraZeneca UK Ltd (Macclesfield, UK). CNBr-activated Sepharose^TM^ 4B beads were purchased from GE Healthcare Bio-Sciences (Uppsala, Sweden). The primary antibodies were from Cell Signaling Biotechnology (Beverly, MA). The antibodies against β-actin and α-tubulin were from Santa Cruz Biotechnology (Santa Cruz, CA) and the Ki-67 antibody was obtained from Thermo Scientific (Fremont, CA). All active proteins were purchased from EMD Millipore Corporation (Temecula, CA). JetPEI poly was from Polyplus-transfection SAS (Saint Quentin Yvelines, France).

### Cell culture

NL20, MRC-5, HCC827, and H1975 human lung normal and cancer cell lines and 293T cells were purchased from American Type Culture Collection (ATCC; Manassas, VA). All cells were cytogenetically tested and authenticated before freezing. All cell culture conditions were performed following ATCC's instructions.

### MTS assay

Cells (1 × 10^3^ per well) for proliferation and cells (5 × 10^3^ per well) for testing compound cytotoxicity were seeded into 96-well plates. After overnight incubation, cells were treated with different concentrations of 244-MPT or 1 μM gefitinib and incubated for 24 or 48 h (cytotoxicity assay) or 24, 48, 72, or 96 h (proliferation assay). CellTiter96 Aqueous One Solution (20 μl; Promega Corporation, Madison, WI) was then added and cells were incubated for another 1 h. Absorbance was read at 492 nm.

### Anchorage-independent cell growth assay

Cells (8 × 10^3^) were suspended in 1 ml BME/10% FBS/0.33% agar with different concentrations of 244-MPT, vehicle or gefitinib and plated on 3 ml of solidified BME/10% FBS/0.5% agar with the same concentration of 244-MPT, vehicle or gefitinib in each well of 6-well plates and cultured for 2 to 3 weeks. Colony numbers were determined by a microscope using Image-Pro Plus software (Media Cybernetics, Inc. Rockville, MD).

### *In vitro* EGFR kinase assay

The *in vitro* EGFR kinase assay was conducted following the instructions provided by Millipore. Active EGFR (100 ng) was mixed with different concentrations of 244-MPT or 1 μM gefitinib. The mixture was incubated with 500 μM angiotensin II for 5 min at room temperature, followed by incubation with 10 μl of an ATP mixture (25 mM MgAc and 0.25 mM ATP containing 10 μCi [γ-^32^P] ATP) for 20 min at 30°C and then 25 μl of reaction mixture were transferred onto P81 papers. The papers were washed with 0.75% phosphoric acid twice and then with acetone once. The radioactive incorporation was determined using a scintillation counter.

### Molecular modeling

The X-ray crystal structure of EGFR wildtype and double mutant was obtained from the RCSB Protein Data Bank [34]. These two structures were prepared under the standard procedure of the Protein Preparation Wizard in Schrödinger Suite 2014 [35]. Hydrogen atoms were added consistent with a pH of 7 and all water molecules were removed. Finally, an ATP binding site-based receptor grid of each kinase was generated for screening. The ZINC natural compound database was prepared using LigPrep of the Schrödinger Suite 2014 utilizing default parameters for screening. Screening was accomplished using the program Glide in Schrödinger Suite 2014 utilizing default parameters under the standard precision (SP) mode followed by the extra precision (XP) mode.

### Energy minimization and molecular dynamics (MD) simulation

To further study the binding of double mutant (L858R/T790M) EGFR with 244-MPT binding at the ATP-binding pocket, a loop energy minimization and MD were conducted in 5 ns by default parameters using the Impact software program from Schrödinger Suite 2014. The binding mode before and after MD was compared.

### Western blot

The protein concentration in cell lysates was determined using a protein assay kit (Bio-Rad Laboratories, Hercules, CA). Lysates were subjected to SDS-PAGE and transferred to polyvinylidene difluoride (PVDF) membranes (EMD Millipore Corporation). Membranes were blocked with 5% non-fat milk and incubated with specific primary antibodies at 4°C overnight. This was followed by incubation with the appropriate horseradish peroxidase (HRP)-conjugated secondary antibody for hybridization. Protein bands were visualized with a chemiluminescence reagent (GE Healthcare Biosciences).

### *In vitro* ATP competitive binding and *ex vivo* pull-down assays

The 244-MPT-conjugated Sepharose 4B beads were prepared according to the manufacturer's protocol (GE Healthcare Biosciences). For the pull-down assay, a 293T cell lysate (500 μg) was incubated with 244-MPT-Sepharose 4B beads or Sepharose 4B beads only (negative control) at 4°C overnight. Binding results were analyzed by Western blotting. For ATP competition assays, an active kinase with different concentrations of ATP was incubated at 4°C overnight. Then 244-MPT–conjugated Sepharose 4B or Sepharose 4B beads (negative control) were added followed by incubation at 4°C for 2 h. The proteins bound to the beads were analyzed by Western blotting.

### Apoptosis assay

Cells (0.75 × 10^6^) were seeded in 60-mm dishes and incubated overnight at 37°C in a 5% CO_2_ incubator and then treated for 24 h with different concentrations of 244-MPT. Then the cells were fixed with ice-cold 70% ethanol at 20°C overnight. After staining with Annexin V, apoptosis was analyzed by 2-color flow cytometry.

### TUNEL assay

Apoptosis was determined using the DeadEnd^TM^ Fluorometric TUNEL System (Promega) according to the manufacturer's instructions. Briefly, after treatment, cells were fixed and disrupted, then equilibrated at room temperature for 5 min, followed by the addition of incubation buffer (45 μl equilibration buffer, 5 μl nucleotide mix, 1 μl rTdT enzyme) and incubated at 37°C for 1 h. The reaction was stopped by incubation with 2 × SSC (300 mM sodium chloride, 30 M sodium citrate) for 15 min at room temperature. After washing, cells were stained with DAPI (1 μg/μl) and the number of TUNEL positive cells was determined by laser scanning confocal microscopy (Nikon C1^S1^ Confocal Spectral Imaging System, Nikon Instruments Co., Melville, NY).

### Xenograft mouse model

Athymic nude mice (6–8 weeks old) were purchased from Charles River and maintained under specific pathogen-free conditions. Mice were divided into 4 groups (*n* = 10 mice per group). The 4 groups were: 1) vehicle control; 2) 150 mg/kg gefitinib; 3) 50 mg/kg 244-MPT; and 4) 200 mg/kg 244-MPT. H1975 human lung cancer cells (2 × 10^6^ in 0.1 ml) were inoculated subcutaneously into the right flank of all mice. Treatment was initiated when tumors reached a mean tumor volume of 25 mm^3^. Treatment with 244-MPT, gefitinib or vehicle control (dimethyl sulfoxide 5% and PEG400 95%) was administered daily by oral gavage. Body weights and tumor measurements were determined 3 times a week, and tumor volume was calculated from measurements of 3 diameters of individual tumors based on the following formula: tumor volume (mm^3^) = length × width × height × 0.52. All studies were performed according to guidelines approved by the University of Minnesota Institutional Animal Care and Use Committee.

### Establishment of the gefitinib-resistant NSCLC PDX model

The gefitinib-resistant NSCLC PDX model was established by exposure to stepwise increasing doses of gefitinib over time. Briefly, PDXs were initiated by subcutaneous implantation of human NSCLC fragments (∼2–3 mm; gefitinib sensitive) coated in Matrigel and implanted through subcutaneous flap incisions in C.B-17 severe combined immunodeflcient (SCID) mice (4–6 weeks old). Mice were treated with gefitinib (10 mg/kg) by oral gavage for 10 to 15 days. Subsequently, PDX-implanted mice were fed a normal diet for 3 to 4 days without gefitinib and then gefitinib was administered by oral gavage (10 mg/kg) another 10 to 15 days. When the xenograft NSCLC tumor reached 500 mm^2^, the mice were sacrificed and the tumors were divided into ∼2–3 mm fragments and implanted into 8 to 10 additional SCID mice for further generations of these tumors. In these 2nd generation gefitinib-treated mice, the gefitinib dose was increased to 50 mg/kg by oral gavage for 10 to 15 days followed by normal diet (no gefitinib treatment) for 3 to 4 days and then the gefitinib treatment by oral gavage (50 mg/kg) was administered for another 10 to 15 days. When these tumors reached 500 mm^3^, the mice were sacrificed and the tumors were divided into ∼2–3 mm fragments and implanted into 8 to 10 additional SCID mice for further generations of these tumors. In these 3rd generation gefitinib-treated mice, the gefitinib dose was increased to 100 mg/kg by oral gavage for 10 to 15 days followed by normal diet (no gefitinib treatment) for 3 to 4 days and then gefitinib treatment by oral gavage (100 mg/kg) was administered for another 10 to 15 days. After three consecutive mouse-to-mouse passages, the gefitinib-resistant NSCLC PDX models became stable and were ready for further study. These studies were approved by the Ethics Committee of Zhengzhou University and the patients whose tumor samples were involved in the study were completely informed and provided consent.

### *In vivo* gefitinib-resistant NSCLC PDX model

After establishing the gefitinib-resistant NSCLC PDX model, we generated enough mice to passage the tumor fragments to 30 mice for 244-MPT treatment in an *in vivo* PDX study. Mice were divided into 3 groups (*n* = 10 mice per group). The 3 groups were: 1) vehicle control; 2) 100 mg/kg gefitinib; and 3) 200 mg/kg 244-MPT. Once the tumor volumes reached approximately 25 mm^3^, mice were treated by oral gavage with vehicle control (dimethyl sulfoxide 5% and PEG400 95%), gefitinib or 244-MPT. Body weights and tumor measurements were performed twice a week and tumor volume was calculated from measurements of 3 diameters of individual tumors based on the following formula: tumor volume (mm^3^) = length × width × height × 0.52. All studies were performed according to guidelines approved by the Zhengzhou University Institutional Animal Care and Use Committee.

### Immunohistochemistry staining

Animal tumor tissues were embedded in paraffin and subjected to immunohistochemistry staining. Tissues were deparaffinized and hydrated and then permeabilized in 0.5% Triton X-100/1 × PBS for 10 min. Immunohistochemical staining for Ki-67 (1:150), phosphorylated (*p*)-EGFR (1:400), *p*-ERK1/2 (1:400) or *p*-Akt (1:50) was performed using the indirect avidin biotin-enhanced horseradish peroxidase method according to the manufacturer's instructions (Vector Laboratories, Burlingame, CA). After developing, all sections were observed by microscope (20x) and analyzed using the Image-Pro Premier software off line (v.9.0) program (Media Cybernetics).

### Statistical analysis

Each experiment was performed 3 times independently. All quantitative data are expressed as mean values ± standard deviation (S.D.) unless otherwise indicated and significant differences were determined by the Student *t* test or one-way ANOVA. A *p* value of < 0.05 was considered statistically significant.

## SUPPLEMENTARY MATERIAL FIGURES


