# Supplementary material for: 244-MPT overcomes gefitinib resistance in non-small cell lung cancer cells
Source: Oncotarget. 2015 Oct 26;6(42):44274–88. doi: 10.18632/oncotarget.6236 (PMC4792556; doi:10.18632/oncotarget.6236)
Supplement: Supplementary file 1 [file oncotarget-06-44274-s001.pdf]

# 244-MPT overcomes gefitinib resistance in non-small cell lung cancer cells

## Supplementary Materials

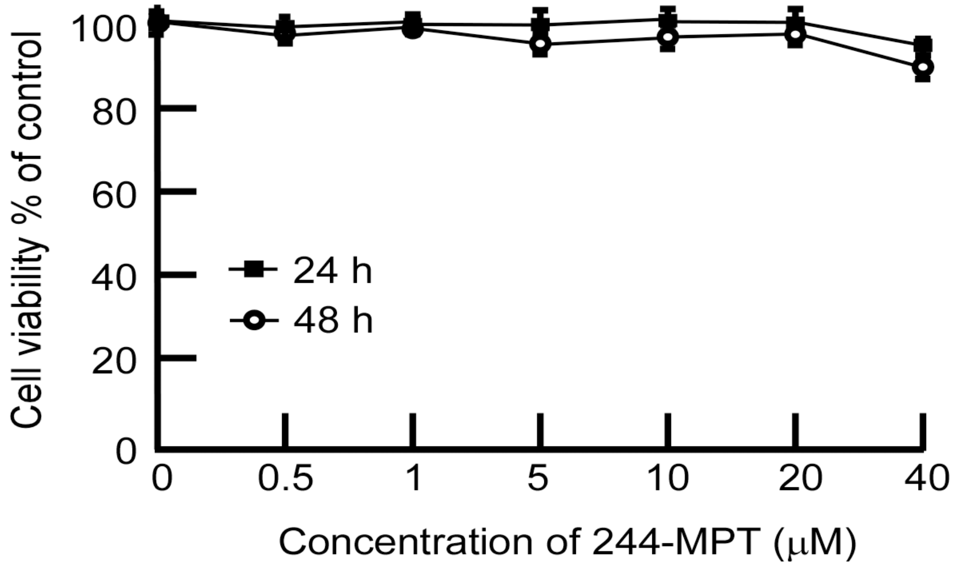


**Supplementary Figure S1: 244-MPT is not toxic to cells up to 40 μM.** Cytotoxicity of 244-MPT was measured by MTS assay in MRC-5 cells, a normal lung cell line. The control group was only treated with 0.1% DMSO, the solvent to dissolve 244-MPT. MRC-5 cells were treated with different concentrations of 244-MPT for 24 or 48 h.

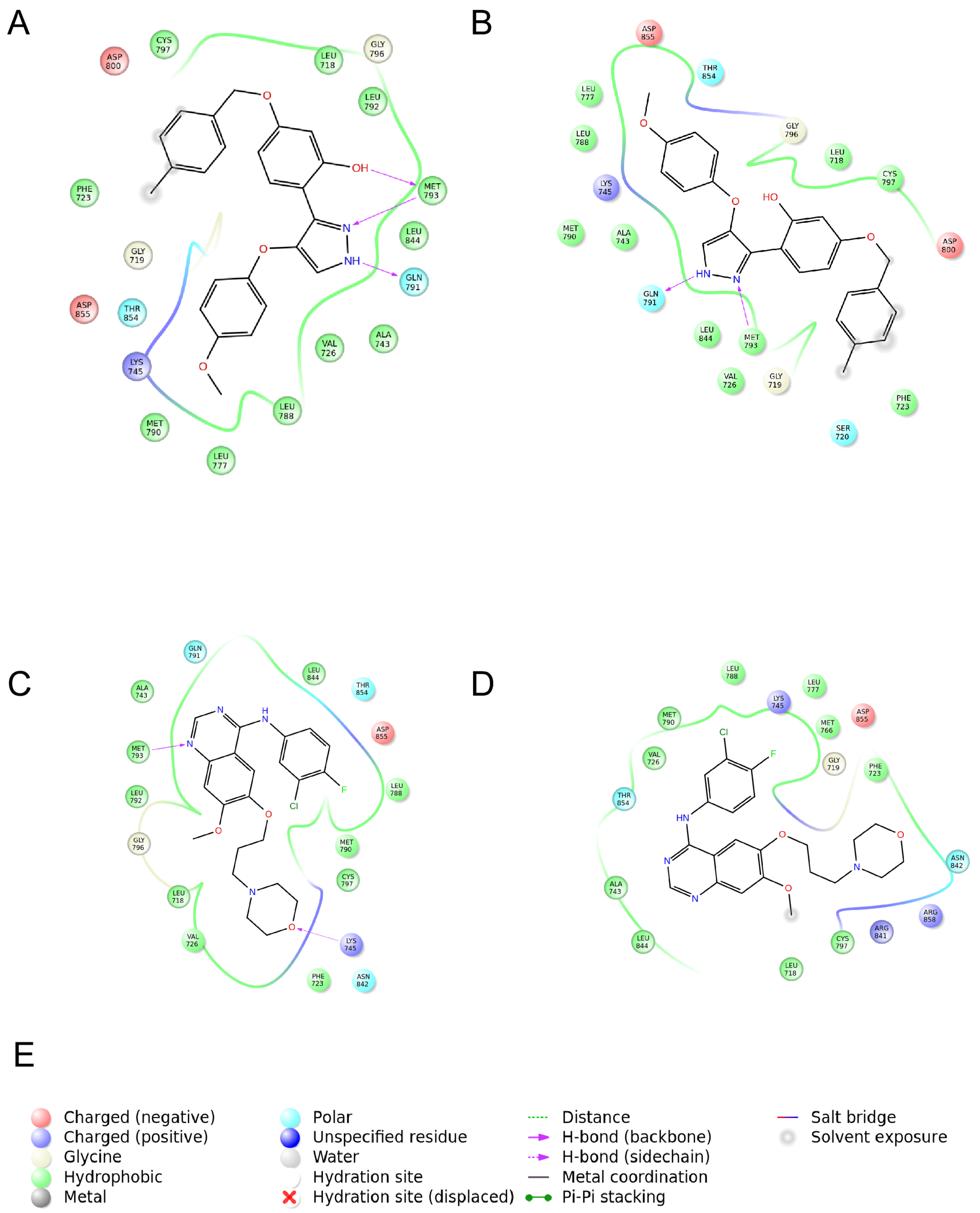


**Supplementary Figure S2: Ligand interaction diagram of double mutant (L858R/T790M) EGFR ATP-binding pocket with 244-MPT or gefitinib after 5 ns molecular dynamics (MD).** Before (**A**) and after (**B**) 5 ns MD binding mode of 244-MPT with double mutant (L858R/T790M) EGFR (See also Supplementary Video S1). Before (**C**) and after (**D**) 5 ns MD binding mode of gefitinib with double mutant (L858R/T790M). (See also Supplementary Video S2). (**E**) Figure legend of interaction diagram.

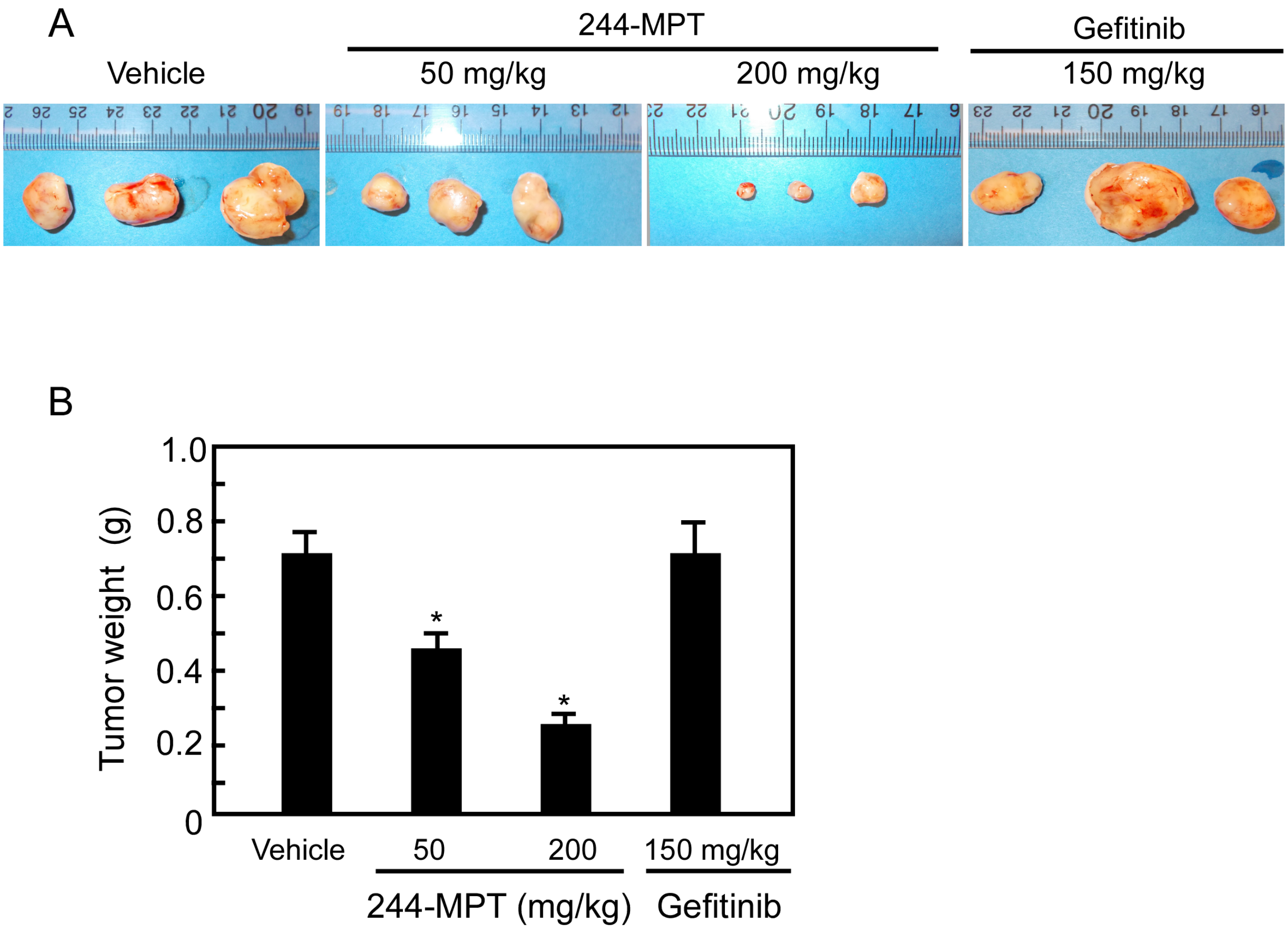


**Supplementary Figure S3: 244-MPT inhibits tumor growth in a gefitinib-resistant NSCLC xenograft mouse model.** After mice were euthanized, (**A**) tumor volume and (**B**) weight were measured. The 244-MPT treated groups exhibited significantly lower tumor volume and weight compared with the vehicle-treated group. Gefitinib treatment had no effect. The asterisk (*) indicates a significant ($p < 0.05$) decrease in tumor weight of 244-MPT-treated mice compared to vehicle-treated mice.
